# Lack of Norovirus Replication and Histo-Blood Group Antigen Expression in 3-Dimensional Intestinal Epithelial Cells

**DOI:** 10.3201/eid1903.121029

**Published:** 2013-03

**Authors:** Melissa M. Herbst-Kralovetz, Andrea L. Radtke, Margarita K. Lay, Brooke E. Hjelm, Alice N. Bolick, Shameema S. Sarker, Robert L. Atmar, David H. Kingsley, Charles J. Arntzen, Mary K. Estes, Cheryl A. Nickerson

**Affiliations:** Author affiliations: University of Arizona, Phoenix, Arizona, USA (M.M. Herbst-Kralovetz, A.L. Radtke);; Arizona State University, Tempe, Arizona (M.M. Herbst-Kralovetz, A.L. Radtke, B.E. Hjelm, A.N. Bolick, S.S. Sarker, C.J. Arntzen, C.A. Nickerson);; Baylor College of Medicine, Houston, Texas, USA (M.K. Lay, R.L. Atmar, M.K. Estes);; Pontificia Universidad, Catolica de Chile, Santiago, Chile (M.K. Lay);; Translational Genomics Research Institute, Phoenix (B.E. Hjelm);; US Department of Agriculture, Dover, Delaware (D.H. Kingsley)

**Keywords:** norovirus replication, 3-D intestinal epithelial cell culture, INT-407, blood group H type antigens, Lewis blood-group system, human intestinal epithelial cells, rotating wall vessel bioreactor, Norwalk virus, viruses

## Abstract

Noroviruses (NoVs) are a leading cause of gastroenteritis worldwide. An in vitro model for NoV replication remains elusive, making study of the virus difficult. A previous study, which used a 3-dimensional (3-D) intestinal model derived from INT-407 cells reported NoV replication and extensive cytopathic effects (CPE). Using the same 3-D model, but with highly purified Norwalk virus (NV), we attempted to replicate this study. Our results showed no evidence of NV replication by real-time PCR of viral RNA or by immunocytochemical detection of viral structural and nonstructural proteins. Immunocytochemical analysis of the 3-D cultures also showed no detectable presence of histo-blood group antigens that participate in NV binding and host tropism. To determine the potential cause of CPE observed in the previous study, we exposed 3-D cultures to lipopolysaccharide concentrations consistent with contaminated stool samples and observed morphologic features similar to CPE. We conclude that the 3-D INT-407 model does not support NV replication.

## Abstract 2

TOC summary: The 3-dimensional intestinal model is not sufficient as a virus replication system for developing vaccines or drugs to control human norovirus infections.

Norovirus (NoV) has been identified as the primary etiologic agent of acute epidemic viral gastroenteritis in industrialized countries (*1*,*2*). Norwalk virus (NV) is the human prototype GI.1 NoV strain; it belongs to the *Caliciviridae* family of positive-sense, single-stranded RNA, nonenveloped viruses (*3*). Human-to-human transmission of NoV occurs primarily through the fecal-oral route, with the small intestine being the initial site of viral replication (*1*,*4*). The lack of an efficient cell culture system in which to study NoV infections has hindered development of antiviral drugs to control or limit NoV outbreaks (*5*).

Currently, animal models are being used to increase our understanding of NoV infections (*6*–*12*); however, no small animal model for NoV mimics the disease manifestations observed in humans (*7**,**11**–**13*). As a result, human outbreaks and volunteer studies have been the primary source for existing knowledge of NV epidemiology and pathogenesis, respectively (*1*,*14*,*15*). A major finding from human NV volunteer studies is that persons with strains containing 2 mutated alleles of the α(1,2) fucosyltransferase (*FUT2*) gene were resistant to NV infection (*16*–*20*). *FUT2* encodes an enzyme that produces histo-blood group antigens (HBGA) on the surface of epithelial cells and in mucosal secretions (*21*,*22*). Persons who lack a functional *FUT2* gene cannot generate ABH antigens in secretions and, thus, are termed nonsecretors (*23**,**24*). HBGA are complex carbohydrates distinguished by different monosaccharides added to a precursor oligosaccharide by fucosyltransferase enzymes (*2*3,*24*). In the human gastroduodenal junction where NV has been shown to bind HBGA type 1 (Lewis b [Le^b^]), is found exclusively on epithelial surfaces, whereas HBGA type 2 is primarily found at the glandular level (*19*,*24*–*26*). In vitro experiments using NV virus-like particles (VLPs) directly showed NV VLP attachment to HBGA, resulting in VLP internalization into the cell (*19*). In addition, NV VLPs were found to preferentially bind to A and H type 1 and Le^b^ carbohydrates (*19*,*27*–*29*). From these critical studies, putative NV receptors were identified, and thus it was hypothesized that a successful in vitro cell culture system would most likely possess these receptors to support NV replication.

Although most of the understanding of NoV infections in humans has been derived from volunteer studies and authentic sporadic outbreaks, extensive volunteer studies have limitations (variability, cost, and institutional review board considerations). As a result, many attempts have been made to develop a reproducible in vitro model system to culture NoV, but none has yet proved successful (*13*,*30*–*33*). However, one publication has reported establishing a productive NoV infection in a 3-dimensional (3-D) organotypic model of human intestinal epithelium derived from the human embryonic intestinal epithelial cell line INT-407 (*30*). We report here an unsuccessful attempt to replicate these findings using NV.

## Materials and Methods

### Cell Lines and NV Stocks

INT-407 cells were obtained from the American Type Culture Collection (Manassas, VA, USA) (CCL-6) and grown in GTSF-2 medium (Hyclone, Logan, UT, USA) containing penicillin/streptomycin and amphotericin B. The human colonic adenocarcinoma epithelial cell line (Caco-2; HTB-37) was obtained from the American Type Culture Collection and was cultured in Earle minimum essential medium, supplemented with 10% fetal bovine serum. All cell cultures were grown at 37°C in 5% CO_2._

NV was purified from the stool specimens of infected volunteers by using a CsCl isopycnic ultracentrifugation gradient as described (*5*). Endotoxin levels were determined by using the Pyrotell Gel-Clot method (Associates of Cape Cod Inc., East Falmouth, MA, USA), performed according to the manufacturer’s protocol (*31*). NV was inactivated by γ-irradiation at doses of 4.25 kGy at room temperature by using a Gammacell-1000 Irradiator (Best Theratronics Ltd., Ottawa, Ontario, Canada) with a cesium-137 source, at a dose rate of 0.5 kGy/h. The titer of NV RNA in each fraction was determined by immunomagnetic capture reverse transcription (RT) PCR as described (*31*,*34*).

### 3-D INT-407 Model of Epithelium of Human Small Intestine 

The 3-D INT-407 model used in these studies was established by using procedures that were as close as possible to those previously described (*30*). In brief, the INT-407 cells were initially grown as monolayers in GTSF-2 medium (Hyclone) containing penicillin/streptomycin and amphotericin B at 37°C in 5% CO_2_ in preparation for seeding into the rotating wall vessel (RWV) bioreactor (*35*). A total of ≈2 × 10^6^ cells were subsequently added to the RWV containing 0.25 mg/mL porous Cytodex-3 microcarrier beads (Sigma-Aldrich, St. Louis, MO, USA) at a ratio of ≈10 cells/bead. Cells were then cultured in the RWV at 18–20 rotations/min as described (*30*,*35*). Fresh medium was replenished every 24–72 h, depending on metabolic activity of cultures. Intestinal aggregates were harvested for use in all studies 28–35 days after they were seeded into the RWV.

### NV Infection

By using a wide-bore pipette (10-mL wide), mature aggregates in 250 μL of GTSF-2 medium were seeded into a 24-well plate (≈1×10^6^ cells/well) and infected with NV at a multiplicity of infection (MOI) of 25 (live or inactivated) for 1 h at 37°C in 5% CO_2_. Plates were rocked every 15 min during the 1-h absorption phase and then overlaid with 750 μL of fresh medium. Wells were incubated for 0-, 6-, 24-, 48-, 72-, or 96-h following infection, imaged, and harvested for RNA extraction or immunofluorescence analysis.

### NV RNA Extraction

RNA was extracted from samples by using the QIAamp Viral RNA Mini Kit (QIAGEN, Valencia, CA, USA). RNA was treated with DNase (Ambion, Foster City, CA, USA) and converted to cDNA by RT by using the iScript cDNA Synthesis Kit (BioRad, Hercules, CA, USA) according to the manufacturer’s instructions. cDNA was stored at −20°C until further analysis.

### Quantitative RT-PCR Analysis

Quantitative RT-PCR (qRT-PCR) was carried out in 25 μL of a reaction mixture containing 2.5 μL of cDNA, 12.5 μL of Supermix (BioRad), a 400 nmol/L concentration of each primer designed to bind to the open reading frame 1 and 2 junction (*36*), and 15 pmol of RING1(a)-TaqMan probe and 5 pmol of RING1(b)-TaqMan probe fluorogenic probes for NV genogroup I (GI) detection. PCR amplification was performed with an iQ5 cycler (BioRad) as described (*36*). For each PCR, an NV GI-specific standard curve was generated by a 10-fold serial dilution (10^9^–10^3^ copies) of purified NV GI cDNA plasmids.

### Microscopy Analysis 

Phase-contrast images were produced on a Zeiss Axiovert 40 CFL microscope (Carl Zeiss, Thornwood, NY, USA) by using Axiovision 4 software (Carl Zeiss) for image processing. For analysis by confocal microscopy, confluent Caco-2 monolayers, INT-407 monolayers, and 3-D INT-407 aggregates were fixed in 4% paraformaldehyde for 30 min at room temperature. Cells were washed and permeabilized with 0.1% Triton X-100 in Dulbecco phosphate-buffered saline (PBS) and blocked at 37°C for 1 h in 4% bovine serum albumin. Cells were stained with anti-H type 1 antibodies (BG-4 [#SIG-313]; Covance, Princeton, NJ, USA; and UEA-1 lectin [L#8146]; Sigma-Aldrich), anti-H type 2–specific monoclonal antibody (Biogenesis; Poole, UK), anti-Le^a^ monoclonal antibody (#4861; Immucor Gamma, Atlanta, GA, USA), and anti-Le^b^ monoclonal antibody (BG-6 [#SIG-3315]; Covance) for 2 h at 37°C at 1:25 dilution. Antiviral protein (VP) 1 and VPg antibodies were used as described (*5*). Unstained control slides and PBS control samples were also used to distinguish background and establish appropriate laser intensity for visualizing experimental slides. Samples were analyzed by using the Zeiss LSM 510 inverted confocal microscope and a 63× objective and Zeiss LSM software.

### *FUT2* Genotyping Assays

DNA was extracted from 200 μL of INT-407 cell pellets by using the QIAamp DNA Blood Mini Kit (QIAGEN). A region of the *FUT2* gene was amplified, by using the following primers: forward 5′-CCCATCTTCAGAATCACCCTGCCGGTGCTG-3′, and reverse 5′-TCGGCCGGCCCGTGGAAACATCCCCAGGTA-3′, which anneal at positions 280–309 and 535–564, respectively, as described (*18*). Results were analyzed as described; in brief, homozygous amino acid at position 428 or TT at position 385 of the *FUT2* gene defines a secretor-negative genotype (*18*,*31*).

### Lipopolysaccharide Treatment

*Escherichia coli* O111:B4 lipopolysaccharide (LPS) (10^6^ EU/mg) was purchased from InvivoGen (LPS-EB; InvivoGen, San Diego, CA, USA). Aggregates were treated with PBS (negative control) or LPS doses ranging from 0.01 to 100.0 μg/mL and incubated for 24 h at 37°C.

### Statistical Analyses

Prism software (GraphPad Software, La Jolla, CA, USA) was used to graph all data and to perform statistical analyses. One-way analysis of variance, followed by Bonferroni post hoc analysis, was used to make statistical comparisons for qRT-PCR analysis. For statistical significance, p<0.05 was considered significant and p<0.01 was considered highly significant.

## Results

### NV Challenge and CPE-like Effects in 3-D INT-407 Aggregates 

**Figure 1:**
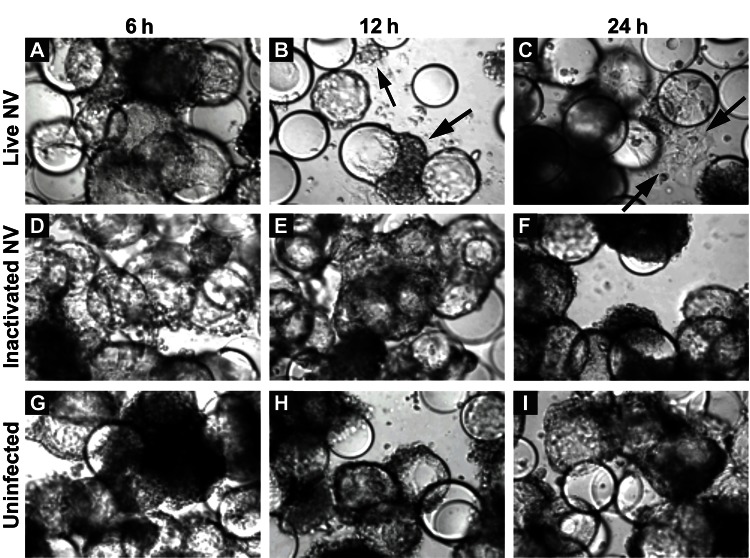
Morphologic changes in Norwalk virus (NV)–infected 3-dimensional INT-407 aggregates. Phase contrast micrographs of 3-dimensional intestinal aggregates cultured in the rotating wall vessel bioreactor and subsequently inoculated with live NV (panels A–C), inactivated NV (γ-inactivated) (panels D–F), or phosphate-buffered saline (mock-uninfected control) (panels G–I) at 6, 12, and 24 h after inoculation. Arrows indicate cells (or cellular debris) that were released from the support beads. The beads appear as large, distinct spheres after the removal of bound cells. Original magnification ×20.

Straub et al. (*30*) previously reported the use of RWV-derived 3-D INT-407 models of small intestinal epithelium for successful NV infection, demonstrated in part by cellular vacuolization and detachment after a 24-h challenge with unpurified extracts obtained from human stool specimens that contained genogroup I or genogroup II NoVs (*30*). To test whether exposure to NV led to gross morphologic changes in 3-D INT-407 aggregates, we prepared a highly purified and concentrated (1.63×10^9^ RNA genomic copies/μL) stock of NV. (Notably, Straub et al. used virus stocks that were clarified by using a 10,000 molecular weight cutoff filter [*30*]). A γ-irradiated NV stock was used as a negative control for NV infectivity and replication because this treatment inactivates virus but preserves the integrity of the virus and the remaining components of the sample (*31*). Three-dimensional INT-407 aggregates were inoculated with live NV or γ-irradiated NV or mock-inoculated with media alone (uninfected) at an MOI of 25 for 6, 12, and 24 h and monitored for potential CPE by light microscopy (Figure 1). Twelve hours post inoculation (hpi), morphologic changes initially attributed to CPE were observed in the 3-D aggregates. These observed morphologic changes included changes in size, shape, and clumping of cells, and detachment of cells from the beads on which the cells had been cultured. In Figure 1, the arrows indicate dissociated cells. Larger distinct circular beads without attached intestinal cells are more abundant in the samples showing damage. These CPE-like morphologic changes were not observed in the uninfected or inactivated samples, which suggests that the NV stock was able to induce gross morphologic changes in the 3-D intestinal aggregates. 

### 3-D INT-407 Aggregates and NV Replication 

**Figure 2:**
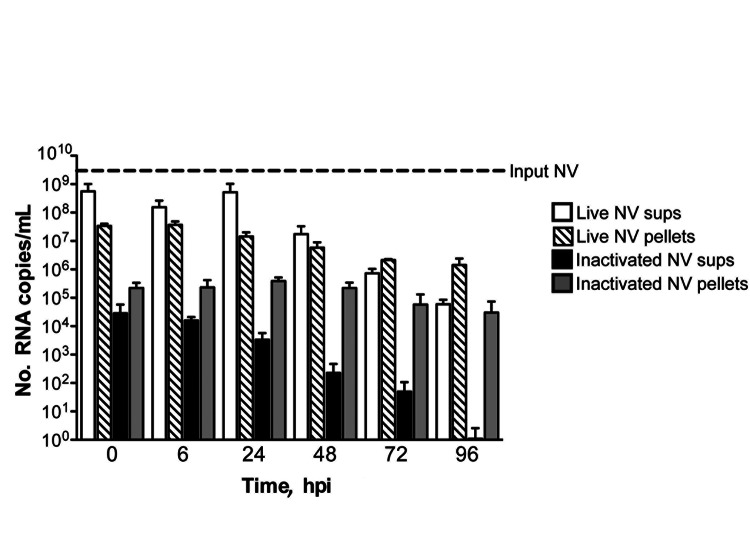
No evidence of productive Norwalk virus (NV) replication in 3-dimensional intestinal aggregates by quantitative reverse transcription PCR analysis. Supernatants (sups) and cell pellets were harvested for RNA at 0, 6, 24, 48, 72, and 96 hours postinoculation (hpi) with live and inactivated NV and analyzed by quantitative reverse transcription PCR. There was no significant increase (p>0.05) in NV RNA copy number over time in the supernatants or cell pellets relative to input virus. Error bars indicate SEM.

To determine whether the CPE-like morphologic changes observed in 3-D INT-407 aggregates were a direct result of NV replication, we performed a kinetic analysis of NV RNA levels by qRT-PCR following NV challenge (Figure 2). The 3-D aggregates were challenged at an MOI of 25 (≈4 × 10^9^ copies of NV total) with live or γ-inactivated NV or mock-challenged with media alone. At each time point (0, 6, 24, 48, 72, and 96 hpi), cellular pellets (containing possible intracellular and cell-associated virus) and supernatants (containing input and released virus) were harvested for RNA extraction, and viral RNA titers were quantified by qRT-PCR. NV RNA copy numbers (indicative of viral replication) did not significantly (p>0.05) increase relative to input copies at any of the time points measured in both cell pellets and culture supernatants (Figure 2). The input virus served as a positive control and for establishing baseline RNA levels for comparison with those of the experimental samples. NV RNA consistently decreased over time in this experiment; however, the differences between these decreases and levels of input virus NV RNA copies over time were not statistically significant for either live or γ-irradiated NV inoculations. These data clearly illustrate that viral RNA levels were not increasing over time as would be expected during a productive viral infection.

**Figure 3:**
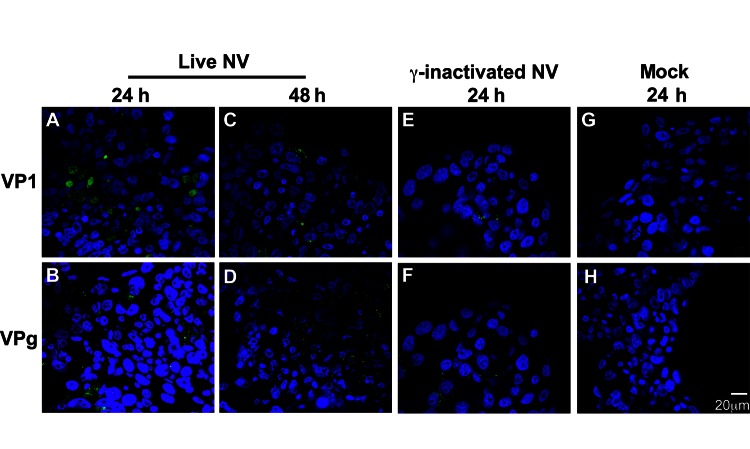
No evidence of productive Norwalk virus (NV) replication in 3-dimensional INT-407 aggregates by confocal microscopy analysis of viral proteins. Three-dimensional INT-407 aggregates 24 h post inoculation (hpi) (panels A, B) and 48 hpi (panels C, D) with live NV, 24 hpi with inactivated NV (panels E, F), or phosphate-buffered saline alone controls (panels G, H). Aggregates were stained for viral capsid protein 1 (VP1) (panels A, C, E, G) or nonstructural protein VPg (panels B, D, F, H). Nuclei were counterstained with 4',6-diamidino-2-phenylindole (DAPI) (not shown in print issue). Original magnification ×63.

To further investigate whether the 3-D aggregates were productively infected, we used confocal immunofluorescence microscopy to analyze 3-D INT-407 aggregates challenged with live NV, γ-irradiated NV, or media alone (mock) and monitored for the presence of NV structural (VP1) and nonstructural (VPg) proteins (Figure 3). During productive NV replication, both structural and nonstructural proteins are detected within cells (*5*). After 24 hpi, VP1 and VPg proteins were detected in the live NV-inoculated aggregates (Figure 3, panels A and B); however, by 48 hpi, evidence for these proteins had markedly diminished and the images resembled the NV-inactivated images (Figure 3, panels C–H). We conclude that the VP1 signal at 24 hpi is most likely a result of input virus and not viral replication, consistent with the qRT-PCR data (Figure 2). The faint VPg signal observed may be nonspecific staining or low-level initial gene expression that does not lead to permissive replication.

### 3-D INT-407 Aggregates and Surface Carbohydrate Tropism 

**Figure 4:**
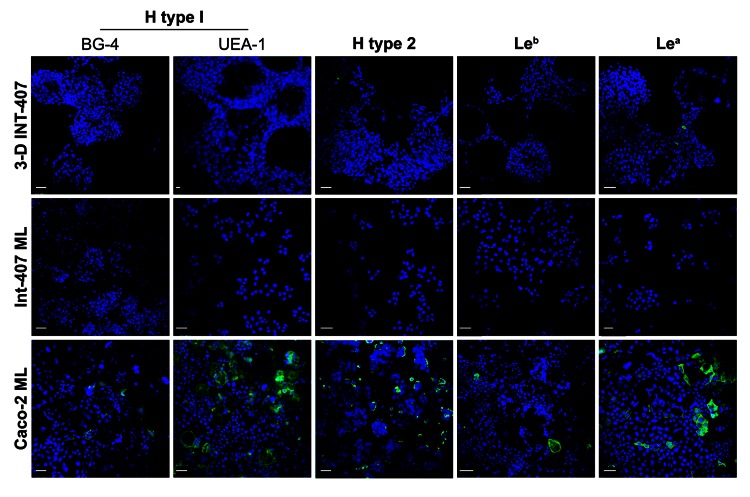
Three-dimensional (3-D) INT-407 intestinal cell aggregates do not express the histo-blood group antigens important for Norwalk virus (NV) attachment. Monolayer (ML) INT-407, 3-D INT-407 aggregates, and Caco-2 ML (positive control) were labeled with 2 different H type 1 (BG-4 and UEA-1) antibodies and with antibodies against H type 2, Le^b^, and Le^a^ (green) and imaged by confocal immunofluorescence microscopy. Nuclei were counterstained with 4',6-diamidino-2-phenylindole (blue). Scale bars = 50 μm.

To better understand why these 3-D intestinal aggregates were not able to productively support NV replication, we characterized them for HBGA expression. NV has been demonstrated to bind to H-type antigens on the surface of mammalian cells, and this binding has been hypothesized to be the mechanism for NV to enter human cells (*5*,*16*,*17*,*19*,*21*). To determine whether 3-D intestinal aggregates contain the *FUT2* gene that converts precursor molecules to H-type antigens (*21*), we performed genotyping analysis on the INT-407 intestinal cells used to produce the organotypic cell culture model. The intestinal cell line was shown to contain the genetic determinants to express a functional *FUT2* (data not shown); therefore, the INT-407 cell line (used to make the 3-D intestinal model) is likely a secretor-positive cell line with the potential to express the H-type receptors necessary for NV binding and, presumably, for infection. However, after performing H-type antigen phenotyping by confocal immunofluorescence microscopy on monolayers and 3-D differentiated intestinal cells, we were unable to detect H type 1 on the surface of the cells by using 2 antibodies specific for different regions of the antigen (Figure 4) (*5*). Further confocal analysis of the INT-407 cells also showed no expression of the other carbohydrate antigens involved in binding of other genogroups of NoV, H type 2, Le^b^, although minimal antigen expression of Le^a^ was observed. Caco-2 cells are shown as a positive control for staining of the surface glycans (*5*). These data demonstrate that both monolayer and 3-D intestinal models lack sufficient expression of the cell surface receptors believed to be required for susceptibility to NV infection.

### LPS and CPE-like Morphologic Changes in 3-D INT-407 Aggregates 

Our data clearly demonstrate that the 3-D intestinal aggregates do not support a productive NV replication; however, we did observe CPE-like morphologic changes after treatment with highly purified NV (Figure 1). One potential cause of these morphologic changes in the 3-D intestinal aggregates could be the presence of contamination of the NV stocks with a bacterial endotoxin, LPS, or other potentially cytotoxic molecules commonly found in human stool samples (the source of virus used in our studies and those of Straub et al. [*30*]). The highly purified virus preparations used in this study were determined to contain low LPS levels, resulting in 10 ng/mL and 1 ng/mL of LPS for live NV and γ-irradiated NV stocks, respectively, added to the aggregates on NV inoculation. LPS is a known cytotoxic inflammation-inducing product; we therefore hypothesized that the contaminating LPS could be a potential cause of the CPE-like morphologic changes observed after NV inoculation. 

**Figure 5:**
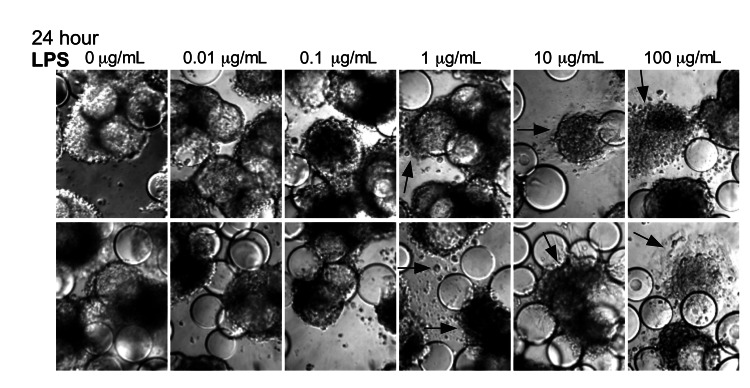
Lipopolysaccharide (LPS) induces morphologic changes consistent with cytopathic effects in Norwalk virus–inoculated 3-dimensional INT-407 aggregates. Two independent sets of light microscopy images show 3-D intestinal aggregates treated with increasing concentrations of LPS for 24 h. Arrows indicate cells (or cellular debris) that were released from the support beads. Original magnification ×20.

To test whether LPS alone induces comparable CPE-like effects at relevant concentrations in the 3-D intestinal aggregates, we treated the aggregates with increasing amounts of LPS (InvivoGen) and monitored for alterations in morphology indicative of cytotoxicity (Figure 5). After 24 h, aggregates treated with a range of LPS concentrations (0.01–100.0 µg/mL) exhibited increasing signs of CPE, as shown by cellular dissociation from the support beads and a partial loss of cell viability (Technical Appendix Figure 1). In comparison with the morphologic changes shown in the technical appendix figure (observed with NV addition to the aggregates), we found that LPS at levels of ≈1 µg/mL was able to induce the CPE-like morphologic changes equivalent to those induced by inoculation of nonpurified NV. Although these levels are higher than the measured level of LPS in the purified NV samples we used, there might also be additional cytotoxic contaminants that were carried from the infected patients’ stool specimens. When these data are taken together, we interpret them as indicating the CPE-like morphologic changes were caused by stool-derived NV samples (Technical Appendix Figure and [*30*]) contaminated with LPS, and perhaps other cytotoxins, and were not a result of virus replication.

## Discussion

Using the same RWV bioreactor cell culture system described by Straub et al. (*30*) to generate well-differentiated 3-D organotypic intestinal epithelial aggregates derived from INT-407 cells, we attempted to validate the published report and further characterize the 3-D intestinal model for NV infection studies. Following a 24-h inoculation of the 3-D INT-407 aggregates with highly purified NV stock, we observed similar morphologic changes to the cells as previously reported (*30*). However, the CPE-like effects observed did not correlate with NV replication, because no measurable increase in viral RNA occurred in a period up to 96 hpi. Rather, CPE-like effects can be mimicked by addition of a known component of human stool samples—LPS. In addition, no increase in viral (structural or nonstructural) proteins could be detected within the aggregates after challenge with NV. Overall, these results support our hypothesis that morphologic changes of the 3-D aggregates after NV inoculation, as observed herein and by Straub et al. (*30*), were most likely a result of LPS within the virus inocula and not due to NV replication.

Our observed lack of HBGA carbohydrates on the surface of the intestinal cells that participate in NV binding to mammalian cells further highlights that this model is likely not a sufficient model system to study NV replication because the cells do not express the putative NV receptor. Previous studies determined that overexpression of FUT2 in natively nonexpressing or low-expressing cell types, or upon differentiation of Caco-2 cells that natively express FUT2, results in a dramatic increase in NV particle binding (*5*,*19*,*32*,*37*,*38*). However, this binding was not sufficient to result in NV infection and replication within these cell types (*5*). Transfecting cells expressing FUT2 with NV RNA (isolated from virus from the stool samples of infected volunteers) did result in NV replication and synthesis of NV proteins within the cells, suggesting that the block in NV replication following virus addition to the cells occurs at a postbinding step and may require an additional cellular factor for viral entry and/or uncoating (*5*,*32*,*38*,*39*). Recently, Lay et al. took a different approach to culture NV: they used primary macrophages and dendritic cells from secretor-positive persons/donors to test their ability to support a NV infection (*31*). Unlike the case with murine NoV, these primary immune cells were unsuccessful in supporting NV infection (*11*,*31*). Future attempts to develop in vitro systems to study NoV replication may include cultures derived from human primary intestinal cells and tissue explants, utilization of Caco-2 cells grown in RWV, use of 3-D intestinal immunocompetent coculture models, or human intestinal organoids derived from pluripotent stem cells (*40*,*41*). These models alone, or in combination, may provide a meaningful way to obtain a system(s) to support productive NoV infection and replication and thus serve as useful tools for studying NoV.

Technical Appendix Figure Three-dimensional INT-407 viability after 24-h lipopolysaccharide (LPS) treatment. The percentage viability of aggregates in Figure 5 was measured by trypan blue exclusion staining. Statistical comparisons were made between treated (0 μg/mL LPS) and LPS-treated aggregates at each concentration using Student *t* test. **Indicates p<0.01.
