# Supplementary material for: Lack of Norovirus Replication and Histo-Blood Group Antigen Expression in 3-Dimensional Intestinal Epithelial Cells
Source: Emerg Infect Dis. 2013 Mar;19(3):431–8. doi: 10.3201/eid1903.121029 (PMC3647661; doi:10.3201/eid1903.121029)
Supplement: Technical Appendix Figure — Three-dimensional INT-407 viability after 24-h lipopolysaccharide (LPS) treatment. The percentage viability of aggregates in Figure 5 was measured by trypan blue exclusion staining. Statistical comparisons were made between treated (0 μg/mL LPS) and LPS-treated aggregates at each concentration using Student t test. **Indicates p<0.01. [file 12-1029-Techapp-s1.pdf]

Article DOI: http://dx.doi.org/10.3201/eid1903.121029

Correlation between Lack of Norovirus Replication and Histo-Blood Group Antigen Expression in 3-Dimensional Intestinal Epithelial Cells

Technical Appendix

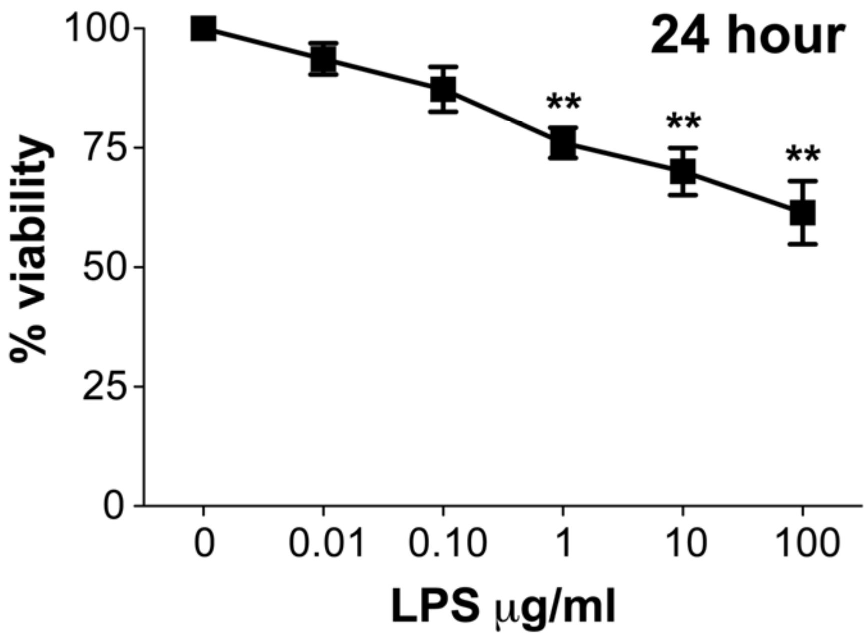

Online Technical Appendix Figure. Three-dimensional INT-407 viability after 24-h lipopolysaccharide (LPS) treatment. The percentage viability of aggregates in Figure 5 was measured by trypan blue exclusion staining. Statistical comparisons were made between treated (0 μg/mL LPS) and LPS-treated aggregates at each concentration using Student *t* test. **Indicates $p<0.01$.
